# Live imaging basement membrane assembly under the pupal notum epithelium

**DOI:** 10.17912/micropub.biology.001105

**Published:** 2024-03-07

**Authors:** Thomas M. Mehaffey, Chloe A. Hecht, James S. White, M. Shane Hutson, Andrea Page-McCaw

**Affiliations:** 1 Dept. Cell and Developmental Biology , Vanderbilt University, Nashville, Tennessee, United States; 2 Program in Developmental Biology , Vanderbilt University, Nashville, Tennessee, United States; 3 Dept. Physics and Astronomy, Vanderbilt University, Nashville, Tennessee, United States; 4 Dept. Biological Sciences, Vanderbilt University, Nashville, Tennessee, United States

## Abstract

Basement membranes are sheet-like extracellular matrices containing Collagen IV, and they are conserved across the animal kingdom. Basement membranes usually line the basal surfaces of epithelia, where they contribute to structure, maintenance, and signaling. Although adult epithelia contact basement membranes, in early embryos the epithelia contact basement membranes only after basement membranes are assembled in embryogenesis. In *Drosophila* , the pupal notum epithelium is a useful model for live imaging epithelial cell behaviors, yet it is unclear when the basement membrane assembles in the pupa, as pupae are undergoing metamorphosis, similar to embryogenesis. To characterize the basement membrane in the pupal notum, we used spinning disk fluorescent microscopy to visualize Collagen IV subunit Vkg-GFP and adherens junction protein p120ctnRFP. Bright punctae of Vkg-GFP were observed in the X-Y plane, possibly representing Vkg-containing cells. We found that a thin continuous Vkg-containing basement membrane was evident at 14 h APF, which became more enriched with Vkg-GFP over the next 6 h, indicating the basement membrane is still assembling during that time. Live imaging of the pupal notum during this time could provide insight into formation, assembly, and repair of the basement membranes.

**Figure 1. Collagen IV increases in intensity in the nascent basement membrane of the pupal notum.**
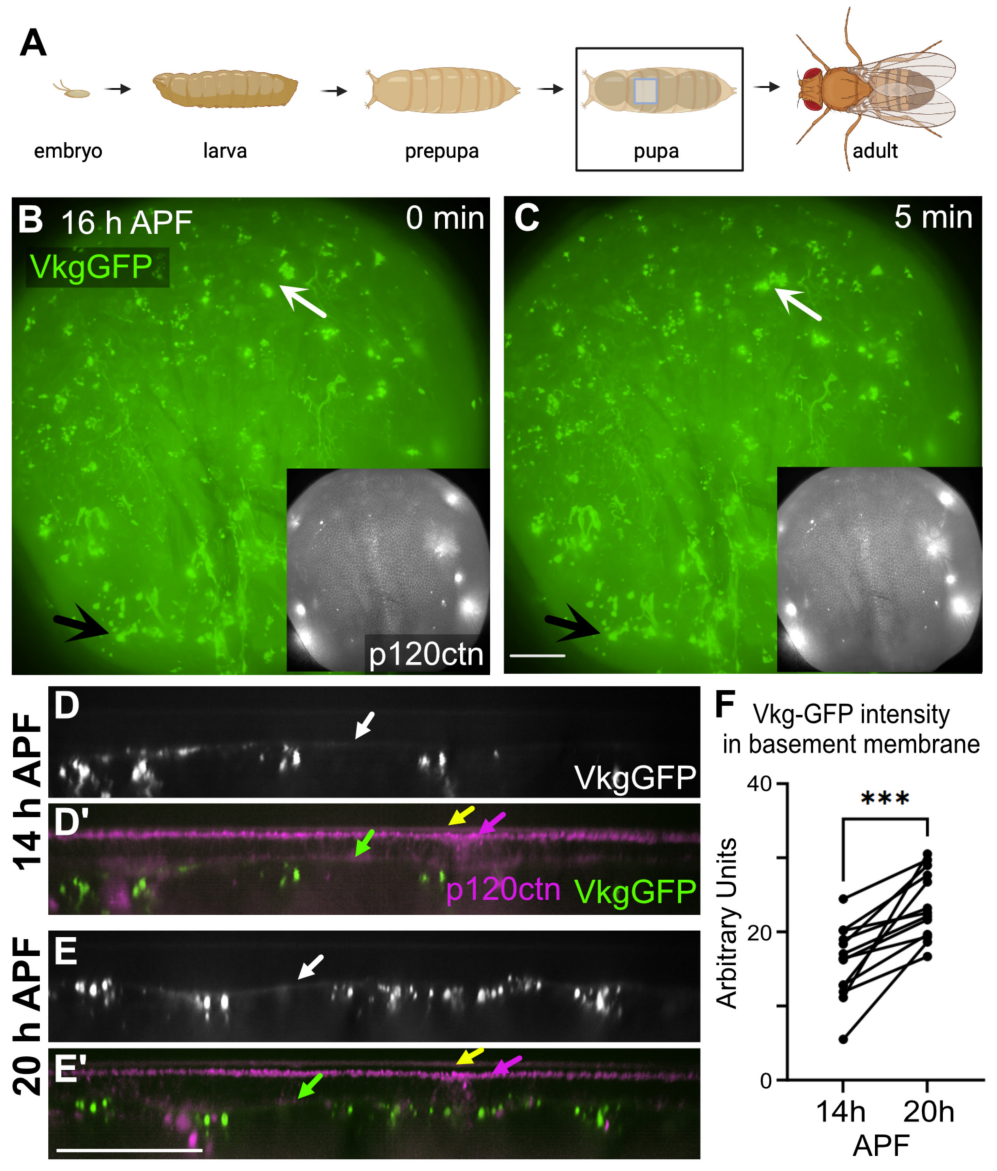
**A.** *Drosophila* life stages. The pupal dorsal notum area imaged is indicated with the blue box in the pupa. **B,C.** Two still images from a movie of a pupal notum, 16 h APF. Vkg-GFP (green) indicates Collagen IV distribution, and p120ctnRFP labels the apical adherens junctions (grey, insets). Many Vkg-GFP punctae are evident. Note that some punctae are immobile over 5 minutes (black arrow), whereas other punctae move within 5 minutes (white arrow). See Extended Data, 20 min movie. Each channel shows a max projection of different optical sections such that the p120ctn image is centered about 11 µm above the Vkg-GFP image. See methods for details of imaging. Anterior is up, bar is 50 µm. **D,E.** A pupal notum imaged in the X-Z axis at 14 h APF and again at 20 h APF, with no intervening imaging. A thin sheet of basement membrane containing Vkg-GFP is visible (white arrows), ~ 10 µm basal to the p120ctn-RFP at the adherens junctions shown in D’ and E’ (magenta arrows). p120ctn-RFP also faintly localizes to the basal cell surface, probably marking the basal spot junctions (Kroeger et al., 2024). Cuticle autofluorescence (yellow arrows) is visible apical to the adherens junctions. **F.** Vkg-GFP intensity increased in basement membranes between 14 h and 20 h APF. 13 paired samples were measured, each from a different pupa, and increases occurred in all of them. p= 0.0037, paired t-test.

## Description

Basement membranes are conserved animal extracellular matrices which underlie the basal surface of epithelia. Basement membranes are composed of highly conserved proteins, and one of the most abundant of these is Collagen IV, which forms a 2D network that gives mechanical support to epithelia (Ramos-Lewis & Page-McCaw, 2018) . Although all adult epithelia are thought to have basement membranes, early embryonic epithelia exist without them until after basement membrane assembly during embryogenesis (Matsubayashi et al., 2017) . In *Drosophila* , after embryogenesis forms the larval body, there is a second morphogenetic developmental phase, metamorphosis, during which the larval tissues are destroyed and new adult tissues are formed ( Fig. 1A ). During these dramatic metamorphic transitions, it is unclear when the adult basement membranes are assembled under epithelia.

The pupal notum has been widely used as a model epithelium because it is amenable to live imaging: the animals are immobile, and after dissection from the pupal case, the notum epithelium is easily visualized through the transparent nascent adult cuticle (O'Connor et al., 2022). Our lab has used the notum as a model for epithelial wound healing ~13-15 h APF (Shannon et al., 2017; O'Connor et al., 2021; Stevens et al., 2023), and it has also been used as a model epithelium to analyze cell extrusion and apoptosis (Marinari et al., 2012; Moreno et al., 2018; Valon et al., 2021) , mitosis (Besson et al., 2015; Pinheiro et al., 2017) , and other epithelial cell behaviors (Guirao et al., 2015) . Despite its popularity as a tissue for basic research in epithelial biology, it is unknown when the basement membrane assembles in this tissue. The notum forms from the imaginal cells in the stalks of wing discs, and these cells expand over the larval epithelial cells, with the pupal notal cells replacing the larval cells as they delaminate and die (Athilingam et al., 2022) . The larval epithelial cells are gone by about 6 h APF, and the pupa can be dissected from the pupal case after ~12 h APF. If the basement membrane assembles after this time, the pupal notum could be a useful model for live-imaging basement membrane assembly; if it has already assembled, it could be a live-imaging model for basement membrane repair after wounding.

To determine whether an epithelial basement membrane was present in the pupal notum epithelium, we used spinning disk fluorescence microscopy to visualize pupae heterozygous for two fluorescent proteins, Vkg-GFP and p120ctnRFP. *Vkg* encodes Col4a2, a constitutive component of all Collagen IV heterotrimers in *Drosophila* . Vkg-GFP is the product of the genomic *Vkg* locus with a GFP-encoded exon inserted near the 5’ end. Because *Vkg-GFP* homozygotes are viable and fertile, the fusion protein is fully functional and expected to localize to all basement membranes. p120ctnRFP is an RFP-tagged component of the adherens junction protein p120ctn, encoded by a transgene expressed ubiquitously by cis-regulatory elements of the *Sqh* gene (Ogura et al., 2018) . Visualizing the nota of 12 h APF flies, we observed obvious tissue movement, obstructing our ability to see the basement membrane, so we looked later at 16 h APF. In an X-Y view, the RFP-labeled adherens junctions were clearly visible ( Fig. 1B,C insets ), but we were not able to detect a layer of GFP-labeled basement membrane. Instead, we observed many bright green punctae of various shapes >10 µm basal to the p120ctn signal ( Fig. 1B,C ). Live imaging over the course of 20 minutes (see Extended Data movie), we observed that some of the punctae were moving (white arrows, Fig. 1B,C ) while some appeared stationary (black arrows, Fig. 1B,C ), and similar punctae were visible as late as 32 h APF. These punctae were not present in nota of control animals not carrying GFP, ruling out autofluorescence. We think it is likely these represent either hemocytes and/or fat body cells, both of which are known to deposit Collagen IV: hemocytes deposit Collagen IV to embryonic tissues (Fessler et al., 1994; Matsubayashi et al., 2017) , and the fat body secretes Collagen IV to many larval tissues (Pastor-Pareja et al., 2011; Ramos-Lewis, LaFever, et al., 2018) . To further attempt to visualize a basement membrane, we projected the red and green channels in the X-Z plane: this revealed a thin line of continuous Vkg-GFP about 10 µm on the basal side of the adherens junctions, constraining the Vkg-containing punctae below it in the body cavity ( Fig. 1D,E ). This thin Vkg-containing sheet appeared to be a nascent basement membrane. To determine whether this low level of basement membrane Collagen IV content was in flux, we imaged a pupae twice, once at 14 h APF and once at 20 h APF, leaving it undisturbed in between with no intermediate imaging to avoid photobleaching. In every one of 13 samples, Vkg-GFP intensity increased between 14-20 h APF ( Fig. 1F ), indicating that this basement membrane is still assembling at this time in metamorphosis.

We conclude that a Collagen IV-containing basement membrane begins to assemble under the notum epithelium before 14 h APF and continues to incorporate more Collagen IV at least through 20 h APF. We speculate that additional Collagen for assembly is secreted by Vkg-GFP containing cells that collect on and move along this basement membrane, evident as the Vkg-GFP punctae we visualized. For Collagen IV to be assembled into basement membranes during embryonic development (Smyth et al., 1999; Urbano et al., 2009) and wound repair (Ramos-Lewis, LaFever, et al., 2018) , laminin must already be present, so it is likely that laminin is present in the assembly pupal basement membrane. This nascent basement membrane may offer an opportunity for live-imaging studies of assembly and/or repair.

## Methods

Fly Husbandry/Cross – Flies used in this study were maintained on cornmeal-molasses media supplemented with dry yeast at 25°C. To allow for basement membrane visualization, virgin females from *Vkg-GFP ^791^* (Flybase ID FBal0211825) stocks were crossed with males of genotype *p120ctnRFP* (Flybase ID FBal0344828). Parents were tossed into new vials every 2-3 days.

Pupae dissection and mounting – Progeny from the *Vkg-GFP ^791^ x p120ctnRFP* were monitored through the transition from larval to pupal stages. Once at the white prepupal stage, the location of each pupa was indicated using a marker and pupae were aged accordingly (see details in O'Connor et al., 2022). Beginning 12 h after puparium formation (APF), pupae were removed from their vials, dissected, and mounted for notum visualization.

Microscopy – 1-2 h after pupal dissection, dissected and mounted pupae were imaged via a Nikon Ti2 Eclipse with X-light V2 spinning disc with a 60X 1.4 NA oil-immersion objective. For initial visualization of the basement membrane in the pupal notum, 40 µm Z-stacks, 0.3 µm intervals, were taken every 5 min over a 20 min period using laser wavelengths to excite GFP and RFP (488nm and 561 nm, respectively), centered just below the epithelial surface. To determine any change in basement membrane morphology and Vkg enrichment during pupal development, 60 µm Z-stacks were taken using the same specified wavelengths once at a 14 h APF and again 6 h later, without disturbing the placement of the samples on the objective.

Fluorescence quantification and analysis – To visualize the basement membrane in the notum most clearly, X-Z plane projections were produced using Volume View in NIS-Elements with 20 µm of depth in the Y plane. For fluorescence quantification of the basement membrane, single X-Z slices were visualized using Slices View on NIS-Elements, and an image was selected in which there was a visibly continuous basement membrane with no occluding punctae; the same slice was re-measured at the later time point. The Binary Editor function was then used to produce a multi-point line which carefully outlined the visible border of the basement membrane according to GFP signal. Using the General Analysis 3 processing tool in NIS-Elements, the Mean Object Intensity in the 488 nm excitation channel was calculated for the multi-point line. To account for the inconsistent background signal between imaging timepoints, secondary multipoint lines were drawn onto the X-Z slices outside of the cuticle boundary, and Mean Object Intensity values were calculated for these lines, representing background fluorescence. For each biological replicate, differences between Basement Membrane Mean GFP Intensity and Background Mean GFP intensity were determined by subtracting the latter value from the former and compared in panel E. Identical X-Z slice locations were used to compare intensity at 14 h APF and 20 h APF. For statistical analysis, a paired t-test was performed in Graphpad Prism, yielding a p-value of 0.0037.

## Reagents

**Table:** 

Short name	Genotype	Creator	Flybase ID
Vkg-GFP	y ^1^ w*; P{PTT-GC}vkg ^CC00791^	Buszczak et al., 2007	FBal0211825
p120ctnRFP	* y w; M{p120ctn ^sqh.TagRFP^ } *	Ogura et al., 2018	FBal0344828

## Extended Data

Description: Live imaging of a pupal notum, 16 h APF, over 20 min. Vkg-GFP (green) indicates Collagen IV. Many Vkg-GFP punctae are evident, some clearly mobile over the 20 min imaging. Max projection over 40 µm Z-depth. Anterior is up.. Resource Type: Audiovisual. DOI: 10.22002/btg83-xwz28
